# Hospital Clerks: Their Duties and Responsibilities

**Published:** 1912-01-06

**Authors:** Herbert J. Dafforne

**Affiliations:** Chief Clerk Leicester Infirmary.


### HOSPITAL OFFICERS: THEIR TRAINING AND WORK.

### I. Hospital Clerks: Their Duties and Responsibilities.

By HERBERT J. DAFFORNE, Chief Clerk Leicester Infirmary.

It has been well said by a veteran London hospital secretary that to become an efficient and useful hospital clerk one of the first essentials, other than good education, address, and knowledge of business, is a cheerful readiness to meet the needs of every case which may arise, while he must at all times be willing to sacrifice himself for the good of the patients and his institution.

In this profession, as in others, if a man takes a keen interest in the work he has to do, gives his whole mind to his duties, and loves his work, he is bound to get on. He must, however, make use of every minute and opportunity that presents itself, never postpone or defer any work which he has to do, for every minute is of value to an efficient hospital officer and the clerk is as much responsible for the work of an institution as any other officer. He must do his share conscientiously and to the best of his ability, for if each individual, never mind what position he or she occupies, does not do so, the smooth working of the institution will not be maintained. In many business houses up and down the country one sees on the wall of the office a card with the words "do it now" in large letters. Each clerk seeking to enter a hospital should have one handed to him on his appointment. Not that the majority of clerks need the reminder, but simply to impress on them the importance of carrying out their part of the institutional work with the least possible delay.

### Training Obtainable in Large Hospitals.

The hospital clerk's duties are most varied, though the larger the institution the more clerks have to be employed, with the result that each has his allotted work and thus, unless promoted from post to post, he does not receive so thorough a general training in the maintenance and management of a hospital as he does his fellow worker who enters a moderate sized institution. As a junior clerk his duties would be mostly ordinary business routine: he would have to copy letters and file all correspondence, summon committee meetings, address envelopes to the subscribers for the annual report, address envelopes for appeals, run messages, issue stationery to the wards, prepare the daily return of the number of patients in each ward so that the secretary may know which wards have vacant beds and which are overcrowded and the actual state of the hospital each morning. He would have to prepare the board-room for all committees, see that the necessary books and stationery are on the table, and also make himself generally useful in the office by helping the other clerks when necessary. As he shows ability in this latter respect and gains a knowledge of other work besides his own he should find the hope of future promotion well or ill-founded.

### The Work of an Out-Patient Clerk.

As an out-patient clerk his duties are perhaps of a still more responsible nature. He has to see that each patient is properly registered, that a case paper is written out for each, that no one is allowed through who is not a fit and proper person to receive free treatment, and if any such persons present themselves, or he has any doubt in his mind about the financial position of any case, it will be his duty to transfer it to the almoner for inquiry. Some institutions have inquiries made into every case; though this may be advisable, it is hardly to be recommended, as the out-patient clerk, after a little practice, can soon tell who is and who is not a suitable case for hospital treatment. Most of our hospitals are largely supported by the Hospital Saturday Funds, and the subscribers to these funds are often the patients or their wives and children, who come for treatment in our out-patient departments and who naturally resent inquiries into their private affairs; and this alone brings the hospital a lot of unpopularity which no institution can afford. The out-patient clerk must treat every patient courteously and kindly, he must remember that he is dealing with sick folk, and often though his temper may be badly tried, he must sacrifice himself for the benefit of the institution and curb any signs of impatience. He must also see that the case papers are properly filed so that they can be obtained with the least possible delay, as it is most important that the doctors whom the patient is under should not be kept waiting while the old notes of the case are being found. The clerk is held responsible for the preparing of all out-patients' figures and has to make a return each day of the number attending the department. He must see that the work of the out-patient porters is carried out satisfactorily and that the patients are treated civilly and with due respect. He must have a thorough knowledge of the rules appertaining to his department and see the same are punctiliously obeyed.

### The Correspondence Clerk's Duties.

As a correspondence clerk his duties, which bring him more in touch with the secretary, are by no means of the lightest. The correspondence of a large institution is surprising, and often no fewer than twenty-five to thirty-five letters are sent out each day. He also has to type personal letters of appeal, acknowledge the various gifts which are showered on our institutions from time to time in the way of periodicals, clothes, fruit, etc., etc. He has to take the minutes down as dictated by the Secretary, and make a draft which, when passed, he has to write up in the minute-books. He has to see that the letters are properly put up, that all enclosures are correct, and the envelopes properly addressed and stamped. For this position it is necessary that the clerk should be an efficient shorthand typist.

*(To be continued.)*

